# Psychoactive Drugs and Quality of Life

**DOI:** 10.1100/tsw.2003.57

**Published:** 2003-08-18

**Authors:** Soren Ventegodt, Joav Merrick

**Affiliations:** The Quality of Life Research Center, Copenhagen K, Denmark

**Keywords:** quality of life, psychotropic drugs, hallucinogenic drugs, substance abuse, health, prevention, public health, Denmark

## Abstract

This study was performed on a representative sample of the Danish population in order to investigate the connection to the use of psychoactive drugs and quality of life (QOL) by way of a questionnaire-based survey. The questionnaire was mailed in February 1993 to 2,460 persons aged between 18 and 88, randomly selected from the CPR (Danish Central Register), and 7,222 persons from the Copenhagen Perinatal Birth Cohort 1959—61.A total of 1,501 persons between the ages 18 and 88 years and 4,626 persons between the ages 31 and 33 years returned the questionnaire (response rates of 61.0% and 64.1%, respectively). Variables investigated in this study were ten different psychotropic drugs and quality of life.Our study showed that over half the Danish population had used illegal psychotropic drugs. The most commonly used was cannabis (marijuana) though experience of this drug appeared not to co-vary with QOL to any significant extent. Cocaine, amphetamine, and psilocybin had been used by 1.2 to 3.3% of the population and this varied with QOL to a clear albeit small extent. LSD has been used by 1.2% of the population and the users had a QOL score 10% lower than those who had never used psychotropic drugs. The group with the lowest quality of life was found to be persons who had used heroin, morphine, methadone, and a mixture of alcohol and tranquilizers (10—20% below the group with the highest quality of life).

## INTRODUCTION

It is generally assumed that the use of euphoric or conscious-altering drugs is detrimental to the quality of life (QOL). However, there are very few studies documented in the literature that have investigated this assumption. For nonclinical populations we could not find research done and studies that have an element of quality of life measurement are restricted to opiate users receiving treatment from various agencies[1,2,3,4,5,6,7].

Due to the scarcity of such studies it is difficult to gain an overall picture of the connection between drug use and quality of life. Furthermore, the quality of life measures that have been used vary considerably, and often the content is so unclear that it is difficult to draw conclusions and to compare studies.

Nonopiate, nonagency samples, though more representative of national patterns of illicit drug use, have been relatively neglected and knowledge on this group is limited.

A better knowledge of the use and misuse of illicit drugs and the effect they have on quality of life is therefore desirable. It is important that the practitioner is aware of the relationship between psychotropic drugs and quality of life, so that he or she may offer suitable help to alleviate the problems presented.

The purpose of the present study was to use the results of a cross-sectional survey examining close to 10,000 Danes to investigate the prevalence of drug use in the Danish population and compare with their quality of life.

## METHODS

### Population 1 (RH)

The Copenhagen Perinatal Birth Cohort 1959–61[8,9,10,11] is a prospective longitudinal perinatal study that included all deliveries (over 20 weeks gestation, birthweight over 250 g) that took place at the University Hospital (Rigshospitalet) in Copenhagen, Denmark during the period of September 21, 1959 to December 21, 1961. The cohort consisted of 9,006 pregnant women who delivered 8,820 live born infants and 362 stillborn or late abortions over 250 g. 170 women gave birth to twins, three to triplets and the remainder were single births. The pregnant women were contacted and examined before delivery as early in pregnancy as possible. In order to evaluate and code the social, medical, and obstetrical information uniformly, all examinations were done by the same physician[8]. He also met with all women after delivery and coded information recorded by the women themseles during pregnancy and also the information describing the events in the delivery room[8].

The infants were examined by one of three pediatricians on the first and fifth days. Physical and neurological examinations were conducted. Upon discharge the mothers received a questionnaire related to child development during the first year, which they brought to the hospital at the age of 1 year for a physical reexamination (a total of 8,425 children survived 28 days)[9,10,11]. Follow-up physical examinations took place at 3 and 6 years with developmental recording done by the mothers between examinations. At a later stage all school health records were retrieved and coded by one physician[12]. More than 1,000 factors relating to pregnancy, birth, and child development were collected on each child resulting in numerous scientific publications over the last 40 years.

In 1993 a new follow-up study was performed and 7,222 of the surviving children were identified (now aged between 31 and 33 years) and contacted with a nonanonymous questionnaire[13]. A written reminder to nonresponders was sent a month later resulting in 4,626 usable responses (f = 2,489, m = 2,131) corresponding to a response rate of 64.1%. The response rate for each individual question was typically a little lower. These 4,626 persons constitute population 1.

## Population 2(CPR)

Using the Danish National Register (CPR-register), 2,460 Danes between the ages 18 and 88 years were chosen randomly and contacted with the same questionnaire[13]. Of the 2,460 contacted we received 1,501 responses, corresponding to a response rate of 61.0%.

## The Questionnaire on Quality of Life

The questionnaire used in the study, "Questionnaire for the Self Evaluation of Quality of Life" (SEQOL), contained 317 questions with 205 in an easy-to-answer multiple-choice format. The questionnaire was divided into the following sections: social information, lifestyle, illness, sexuality, self-perception, life perception, and eight series of questions measuring the quality of life[13].

The development of the questionnaire and progression of the study was guided by the following methodological demands for quantitative questionnaire-based quality of life research[13]:

1. A clear definition of the quality of life.
2. A philosophy of life based on the definition of point 1.
3. A theory that has this philosophy as its framework by
    a) deducing questions that are unambiguous, mutually exclusive, and together are fully exhaustive, and by
    b) establishing the relative weighting of each question.
4. A number of response options that might be given a quantitative interpretation on a fraction scale.
5. Technical checks in terms of reproducibility, sensitivity, well-scaledness, etc.
6. The survey must be meaningful to both researchers, respondents, and those who use the results.
7. An appreciation of the aesthetic dimension.

The present study followed these requirements[13] with the theoretical basis for quality of life measurement based upon the integrative quality of life theory[13]. It organises eight individual theories of quality of life into a spectrum ranging from subjective (self-evaluated) to objective (externally evaluated) quality of life and spanning a core of theories that consider quality of life as deriving from human nature or human existence itself (existential theories). These eight theories or dimensions of life quality were operationalized into eight rating scales, then grouped into three dimensions:

**I. Subjective Dimensions**

1. Immediate, self-experienced well-being
2. Satisfaction with life
3. Happiness

**II. Existential Dimensions**

4. Needs fulfillment
5. Subjective experience of objective temporal domains (family, work, leisure)
6. Subjective experience of objective spatial domains (satisfaction with social relationships)

7. Expression of life's potentials

**III. Objective Dimension**

8. Objective factors (income, employment, education, etc.)

Eighty-five of the questions in the questionnaire were used to measure quality of life along these eight dimensions. A Likert-scale with five response options was symmetrically arranged around a neutral midpoint. As an example, well-being was measured by the question, "How are you feeling now?", and the response options given were "very good," "good," "neither good nor poor," "poor," and "very poor." The central and precisely worded mid-point (neither good nor poor), the response options symmetrically aligned up and down the scale (good, poor) and the use of the same amplifier (very) all combine to suggest that the five points on the scale may be considered equidistant.

If an underlying scale was selected ranging from 0 to 100%, from the worst imaginable to the best imaginable quality of life, the five response options may be reasonably positioned at 10, 30, 50, 70, and 90%. In other words, if a respondent checks "good," his or her well-being is measured as 70%. In this manner, an approximated ratio scale was obtained, so that means could be computed and compared.

A weighted mean for the eight quality of life dimensions was computed by subjective and existential measures, respectively. The resulting overall measure was global (covers all aspects of life, not merely health-related aspects) and generic (not disease-related or intended for a specific category of patients).

Significance levels for the relationships between each variable and the measured quality of life were computed for the continuous variable using classical correlation and a modified regression[13], while in the case of the discrete variables, every group was tested individually against the rest of the sample HO: $\mu i = \mu non\text{-}i$ (that is, the null hypothesis that the mean quality of life for a particular group [for example, smokers] was significantly different from the mean quality of life for the rest of the population [for example, those who have used cocaine ].

The questionnaire has been validated[13,14,15,16], and the measurement instruments (the rating scales) proved to be valid and sensitive to the same degree as commonly recognised international instruments. A 1-month and a 3-month test-retest for reproducibility showed correlation coefficients for the eight instruments ranging from 0.6 to 0.9. A qualitative assessment of the validity of the questionnaire was performed, in which 80% of the respondents indicated that the questionnaire items expressed all dimensions relating to their quality of life, 17% were in doubt, and 3% felt they did not express all dimensions, which was found to be acceptable.

## The Questionnaire on Drugs

The question-measures on the use of psychotropic drugs were multiple choice, where the respondent could mark an earlier or present use of the following drugs:

| | | | |
|---|---|---|---|
| 1 | hashish/cannabis/marijuana | 7 | crack |
| 2 | LSD | 8 | ecstasy |
| 3 | psilocybin (mushrooms) | 9 | methadone |
| 4 | mescaline (cactus) | 10 | heroin, morphine |
| 5 | amphetamine, speed | 11 | tranquilizers with alcohol |
| 6 | cocaine | | |

For the discrete variable (e.g., use of a given drug) the average quality of life of the group that has used the drug was compared with the average quality of life of population or cohort as a whole.

(Even though, in this isolated case, it would have been more suitable to compare with those that do not have the problem, the above comparison was chosen to allow comparisons with correlations of other variables examined in this study.)

The variation over the measured interval is given both as an absolute number and a percentage of the population's or the cohort's average quality of life. This number has the advantage that it can be compared from variable to variable, such that the size of the correlation between quality of life and a specific variable (e.g., an experience of cannabis) can be compared with the size of the correlation between quality of life and other variables (e.g., an experience of cocaine).

These varying degrees of correlations between quality of life and different measured variables are divided into five classifications, which qualitatively compensates for the possibility that the measured variable, directly or indirectly, forms part of one or more of the eight quality of life measures as well as possible scaling problems. The observed deviation in the size of these correlations translates into the following categories: "very small" (responses corresponding to a correlation of 0 to 5%), "small" (5 to 10%), "intermediate" (10 to 20%), "large" (20 to 40%), and "very large" (over 40%). Please note that the number of crack and ecstasy users in 1993 were too small to give significant measures for these groups.

## RESULTS

Hallucinogens became extremely popular in Denmark during the youth revolution of the 1960s, and the use of cannabis among the young, despite legislation making these drugs illegal, is believed to be almost as common as tobacco and alcohol. Hallucinogens are characterised by their wide-ranging psychological effects. They vary in strength from the mild such as cannabis to the strong such as psilocybin or LSD.

### Cannabis/Hashish/Marijuana

Cannabis use in Denmark, like alcohol, tends to be recreational. The dose price ratio for this illegal drug is often considerably lower than the dose price ratio for alcohol. 55.7% of the 31 to 33 year olds (RH) and 24.9% of the population sample (CPR) had used cannabis (see Tables 1 and 2). Among the 31 to 33 year olds those that had used cannabis had a QOL score 4.0% below the cohort's mean QOL score, while for the population sample, those that had used cannabis had a QOL score 2.8% below the general population mean QOL score; this is a small but significant correlation. Tables 3 and 4 show that the correlation is consistent through the eight quality of life measures.

### LSD (Lysergic Acid Diethylamide)

Since its emergence in the 1960s as a cult drug, LSD has typically been used by the experimental youth of Denmark as a chemical short-cut to transcendental experience and introspection or as an escape from the established values and behaviour patterns of society.

3.0% of the 31 to 33 year olds and 1.2% of the population sample had used LSD (see Tables 1 and 2). The 31 to 33 year olds who had used LSD had a QOL score 11.6% below the cohort average, while for the population sample it was 9.5% below population average, which again is a small but a significant correlation. Tables 3 and 4 demonstrate that the connection was uniform through all eight quality of life measures with a tendency to be bigger in the objective measure for the 31 to 33 year olds.

**TABLE 1**
**Quality of Life at Ages 31 to 33 Years vs. Experience of a Given Drug***

| | % | 1 | | 2 | | 3 | | 4 | | 5 | | 6 | | 7 | | 8 | | Overall QL (Weighted) | Test (p–value) |
|---|---|---|---|---|---|---|---|---|---|---|---|---|---|---|---|---|---|---|---|
| No Drugs | 42.9 | 73.2 | 1979 | 71.0 | 1978 | 67.7 | 1979 | 69.8 | 1818 | 76.3 | 1952 | 69.1 | 1909 | 63.1 | 1966 | 74.9 | 1936 | 71.7 | ** 0.0001 |
| Cannabis | 55.7 | 71.1 | 2570 | 68.2 | 2571 | 65.1 | 2567 | 67.2 | 2349 | 73.9 | 2534 | 65.6 | 2502 | 60.1 | 2563 | 71.9 | 2516 | 68.9 | ** 0.0001 |
| LSD | 3.0 | 67.6 | 141 | 62.5 | 141 | 62.1 | 141 | 65.4 | 123 | 73.0 | 133 | 60.9 | 136 | 54.6 | 139 | 63.1 | 134 | 63.5 | ** 0.0001 |
| Psilosybin | 5.1 | 69.2 | 234 | 66.2 | 234 | 63.2 | 234 | 65.9 | 205 | 73.8 | 225 | 62.0 | 225 | 56.7 | 232 | 65.5 | 225 | 65.4 | ** 0.0001 |
| Amphetamine | 14.3 | 69.0 | 662 | 65.3 | 662 | 62.9 | 662 | 65.1 | 596 | 72.7 | 644 | 62.8 | 640 | 57.4 | 656 | 67.0 | 642 | 65.8 | ** 0.0001 |
| Cocaine | 6.0 | 68.8 | 279 | 66.3 | 279 | 64.1 | 279 | 66.9 | 245 | 74.1 | 269 | 63.4 | 267 | 57.4 | 277 | 67.3 | 267 | 66.4 | ** 0.0001 |
| Crack | 0.1 | 50.0 | 5 | 50.0 | 5 | 38.0 | 5 | 48.1 | 4 | 48.1 | 4 | 55.0 | 4 | 51.0 | 4 | 60.7 | 4 | — | — |
| Ecstasy | 0.3 | 68.6 | 15 | 62.0 | 15 | 59.3 | 15 | 63.4 | 14 | 70.4 | 14 | 59.4 | 14 | 55.9 | 14 | 64.6 | 14 | — | — |
| Methadone | 1.4 | 62.2 | 64 | 59.7 | 64 | 59.1 | 64 | 64.8 | 53 | 71.8 | 56 | 60.7 | 58 | 53.6 | 62 | 56.8 | 61 | 59.9 | ** 0.0001 |
| Heroine, morphine | 2.8 | 63.5 | 130 | 61.2 | 130 | 58.9 | 130 | 64.4 | 106 | 72.5 | 121 | 61.1 | 121 | 55.4 | 128 | 61.9 | 119 | 62.2 | ** 0.0001 |
| Tranquilizers and alcohol | 4.7 | 62.4 | 218 | 59.6 | 218 | 58.4 | 218 | 61.3 | 187 | 68.7 | 207 | 59.4 | 210 | 54.0 | 215 | 60.5 | 202 | 60.5 | ** 0.0001 |
| Overall average; Total number | | 70.7 | 6277 | 68.0 | 6277 | 65.1 | 6274 | 67.5 | 5682 | 74.3 | 6141 | 65.7 | 6068 | 60.0 | 6238 | 71.0 | 6102 | 68.6 | |

*The columns show: the percentage who tried the drug mentioned; the average QOL Score (on a 0–100 scale), the number of respondents in each group; the overall QOL score and resulting p value.

Columns: 1 – Immediate, self–experienced well–being; 2 – Life satisfaction; 3 – Happiness; 4 – Fulfillment of needs; 5 – Experience of objective, temporal domains (family, work, leisure); 6 – Experience of objective, spatial domains (self, others, world); 7 – Expression of life's potentials; 8 – Objective factors

## Mescaline

Mescaline is found in a variety of hallucinogenic cacti such as peyote and Don Pedro and was traditionally used by native Americans for medicinal and ceremonial purposes. In Denmark it exists as an illegal synthetic drug, and is used in much the same way as LSD, though it is said to give a "softer" and more "bodily" experience.

1.2% of the 31 to 33 year olds and 0.2% of the population sample had used mescaline (see Tables 1 and 2). The group of 31 to 33 year olds that had used mescaline had a QOL score 11.6% below the cohort's average score.

## Psilocybin

It is now common knowledge that a number of wild Danish mushrooms contain the hallucinogenic agent psilocybin. Psilocybin mushrooms, traditionally used by native Americans, are used in Denmark for much the same purpose as LSD. This research together with that of others[17] suggested that psilocybin is now the most frequently used hallucinogenic in Denmark. One explanation for this may be the common perception that the psilocybin trip is less dangerous for the psychological balance.

**TABLE 2**
**Quality of Life of the CPR Group vs. Experience of a Given Drug***

| | % | 1 | | 2 | | 3 | | 4 | | 5 | | 6 | | 7 | | 8 | | Overall QL (Weighted) | Test (p–value) |
|---|---|---|---|---|---|---|---|---|---|---|---|---|---|---|---|---|---|---|---|
| No Drugs | 74.5 | 72 | 1083 | 70 | 1079 | 66 | 1081 | 70 | 958 | 77 | 1020 | 70 | 976 | 46 | 1070 | 70 | 982 | 68.3 | ** 0.0001 |
| Cannabis | 24.9 | 71 | 367 | 68 | 367 | 65 | 367 | 67 | 338 | 74 | 359 | 66 | 352 | 44 | 367 | 68 | 352 | 66.5 | ** 0.0001 |
| LSD | 1.2 | 65 | 17 | 62 | 17 | 58 | 17 | 58 | 15 | 71 | 16 | 64 | 16 | 41 | 17 | 66 | 17 | 61.9 | ** 0.0001 |
| Psilosybin | 1.2 | 74 | 18 | 63 | 18 | 63 | 18 | 67 | 16 | 73 | 18 | 64 | 17 | 42 | 18 | 69 | 18 | 65.9 | ** 0.0001 |
| Amphetamine | 3.3 | 74 | 49 | 70 | 49 | 65 | 49 | 68 | 48 | 75 | 49 | 66 | 45 | 44 | 48 | 71 | 46 | 68.1 | ** 0.0001 |
| Cocaine | 1.2 | 66 | 17 | 61 | 17 | 57 | 17 | 58 | 16 | 70 | 17 | 60 | 16 | 38 | 17 | 57 | 17 | 58.3 | ** 0.0001 |
| Methadone | 0.4 | 63 | 6 | 60 | 6 | 57 | 6 | 54 | 5 | 71 | 6 | 55 | 6 | 33 | 6 | 54 | 6 | 55.7 | ** 0.0001 |
| Heroine, morphine | 0.5 | 73 | 6 | 67 | 6 | 50 | 6 | 57 | 6 | 67 | 5 | 62 | 5 | 42 | 6 | 65 | 6 | 61.7 | ** 0.0001 |
| Tranquilizers and alcohol | 0.5 | 67 | 7 | 61 | 7 | 53 | 7 | 58 | 7 | 72 | 6 | 61 | 6 | 38 | 7 | 49 | 6 | 55.6 | ** 0.0001 |
| Overall average; Total number | | 72.1 | 1570 | 69.2 | 1566 | 65.3 | 1568 | 68.9 | 1409 | 75.8 | 1496 | 68.9 | 1439 | 44.9 | 1556 | 69.0 | 1450 | 67.5 | |

*The columns show: the percentage who tried the drug mentioned; the average QOL Score (on a 0–100 scale), the number of respondents in each group; the overall QOL score and resulting p value.

Columns: 1 – Immediate, self–experienced well–being; 2 – Life satisfaction; 3 – Happiness; 4 – Fulfillment of needs; 5 – Experience of objective, temporal domains (family, work, leisure); 6 – Experience of objective, spatial domains (self, others, world); 7 – Expression of life's potentials; 8 – Objective factors

5.1% of the 31 to 33 year olds had used psilocybin compared with 1.2% of the population sample (see Tables 1 and 2). Those 31 to 33 year olds who had used psilocybin had a QOL score 8.9% below the cohort mean, while the population group for those who had taken psilocybin was 3.7% below the mean QOL score, which we classify as a small but significant connection. Tables 3 and 4 show that the connection is uniform through all quality of life measures with a tendency to be a little larger in the objective measure for the 31 to 33 year olds.

## Stimulants

While hallucinogenic drugs alter experience, the stimulants work only to amplify feelings and/or mental activity. Ecstasy is placed on the borders between these two, while the normal course of intoxication is said to be a temporary amplification of feelings and hallucinations can be experienced.

## Amphetamines

Amphetamines have a profoundly stimulating and arousing effect on the central nervous system. In Denmark it is typically used by the young, who want extra energy and an enhanced state of mood at social occasions. Amphetamines are significantly cheaper than cocaine on the street, but the effect is said to be almost the same except that the amphetamine trip lasts about 10 times as long.

**TABLE 3**
**Quality of Life at Ages 31 to 33 Years vs. Experience of a Given Drug**

| | % | 1 | 2 | 3 | 4 | 5 | 6 | 7 | 8 | Overall QL (Weighted) | Test (p–value) |
|---|---|---|---|---|---|---|---|---|---|---|---|
| No Drugs | 42.9 | 1.8 | 2.3 | 2.3 | 2.2 | 1.9 | 3.0 | 2.8 | 2.4 | 2.8 | ** 0.0001 |
| Cannabis | 55.7 | –1.2 | –1.6 | –1.6 | –1.6 | –1.4 | –2.3 | –2.1 | –1.7 | –2.1 | ** 0.0001 |
| LSD | 3.0 | –6.0 | —9.9 | –6.2 | –4.3 | –2.6 | –9.2 | –11.0 | –13.8 | –11.0 | ** 0.0001 |
| Psilosybin | 5.1 | –3.7 | –4.5 | –4.5 | –3.6 | –1.4 | –7.6 | –7.7 | –10.4 | –7.7 | ** 0.0001 |
| Amphetamine | 14.3 | –4.0 | –5.8 | –5.0 | –4.8 | –2.9 | –6.4 | –6.5 | –8.3 | –6.5 | ** 0.0001 |
| Cocaine | 6.0 | –4.3 | –4.3 | –3.2 | –2.1 | –1.1 | –5.5 | –6.5 | –8.0 | –6.5 | ** 0.0001 |
| Crack | 0.1 | –30.5 | –27.9 | –42.6 | –29.6 | –26.6 | –24.0 | –26.9 | –17.0 | –26.9 | — |
| Ecstasy | 0.3 | –4.5 | –10.6 | –10.3 | –7.2 | –5.9 | –11.4 | –9.0 | –11.6 | –9.0 | — |
| Methadone | 1.4 | –13.5 | –13.9 | –10.7 | –5.2 | –4.2 | –9.6 | –12.8 | –22.3 | –12.8 | ** 0.0001 |
| Heroine, morphine | 2.8 | –11.6 | –11.7 | –10.9 | –5.7 | –3.2 | –9.0 | –9.8 | –15.4 | –9.8 | ** 0.0001 |
| Tranquilizers and alcohol | 4.7 | 4.7 | –14.0 | –11.7 | –10.3 | –8.2 | –11.4 | –12.0 | –17.3 | –12.0 | ** 0.0001 |
| Overall average; Total number | 42.9 | 1.8 | 2.3 | 2.3 | 2.2 | 1.9 | 3.0 | 2.8 | 2.4 | 2.8 | |

The columns show: the percentage who tried the drug mentioned, the deviation from average QOL score in the measures 1-8; the deviation from the overall QOL score; the resulting p-value.

14.3% of the 31 to 33 year olds and 3.3% of the population sample had used amphetamines. Of the 31 to 33 year olds who had used amphetamines, 8.4% had a QOL score below the cohort mean QOL, and 0.7% of the population group had a QOL score below the mean, which is a small but important and significant connection. Tables 3 and 4 show that the connection is consistent through all eight QOL measures.

The results do not strongly support the hypothesis that amphetamines are detrimental to quality of life. Physicians and social welfare agencies should, however, be aware of the use of amphetamines by the young to boost low self-confidence in social situations. Low self-confidence is statistically linked to low quality of life[18,19] and therefore can be an explanation of the correlation.

## Cocaine

Cocaine has been used as a stimulant by the Indians of Central America. Like amphetamines, it gives an enhanced sense of mental and physical energy and elevates the mood, but is — probably because of the high price — a lot less prevalent.

Of the 31 to 33 year olds 6% had used cocaine, while only 1.2% of the population sample had used cocaine (see Tables 1 and 2). Of the 31 to 33 year olds who have used cocaine, 7.6% had a QOL score below the average for the cohort, while for the population sample, those who had used cocaine had a QOL score 15% below the average. This is a small to intermediate, significant connection. In Tables 3 and 4 it is shown that the connection is uniform through all eight measures, though there is a tendency for the connection to be bigger in the objective measure.

The results supported, to a degree, the hypothesis that cocaine was detrimental to quality of life, although it is not known if the low QOL leads to the use of cocaine.

**TABLE 4**
**Quality of Life of the CPR Group vs. Experience of a Given Drug**

| | % | 1 | 2 | 3 | 4 | 5 | 6 | 7 | 8 | Overall QL (Weighted) | Test (p–value) |
|---|---|---|---|---|---|---|---|---|---|---|---|
| No Drugs | 75 | 0.5 | 0.9 | 0.7 | 1.0 | 1.0 | 1.6 | 1.4 | 0.6 | 0.9 | ** 0.0001 |
| Cannabis | 25 | –0.8 | –2.1 | –0.8 | –2.8 | –2.4 | –4.1 | –2.9 | –1.3 | –1.91 | ** 0.0001 |
| LSD | 1 | –9.4 | –11.1 | –11.1 | –15.9 | –6.2 | –8.3 | –9.9 | –5.2 | –8.6 | ** 0.0001 |
| Psilocybin | 1 | 3.3 | –8.8 | –3.3 | –3.3 | –4.5 | –6.9 | –7.4 | 0.1 | –2.8 | ** 0.0001 |
| Amphetamine | 3 | 2.8 | 0.8 | –1.2 | –1.7 | –0.9 | –5.2 | –1.7 | 3.1 | 0.5 | ** 0.0001 |
| Cocaine | 1 | –7.7 | –12.8 | –12.9 | –15.9 | –7.4 | –13.2 | –15.5 | –18.1 | –14.1 | ** 0.0001 |
| Methadone | 0.4 | –12.1 | –13.6 | –13.5 | –22.1 | –6.4 | –20.6 | –26.2 | –22.4 | –18.1 | ** 0.0001 |
| Heroine, morphine | 0.5 | 1.8 | –4.0 | –23.6 | –18.3 | –11.4 | –10.5 | –7.1 | –6.2 | –8.9 | ** 0.,0001 |
| Tranquilizers and alcohol | 0.5 | –6.8 | –11.6 | –19.3 | –17.1 | –5.0 | –11.5 | –14.8 | –29.3 | –18.0 | ** 0.0001 |
| Overall average; Total number | 42.9 | 1.8 | 2.3 | 2.3 | 2.2 | 1.9 | 3.0 | 2.8 | 2.4 | | |

The columns show: the percentage who tried the drug mentioned, the deviation from average QOL score in the measures 1-8; the deviation from the overall QOL score; the resulting p-value.

Cocaine, as with amphetamines, is used mainly by the young to boost self-confidence in social situations. Low self-confidence is directly correlated to a lower quality of life[18,19], and this could be a possible explanation for the connection found with quality of life. This may mean that drugs such as cocaine are in themselves not dangerous, but their use functions as an indicator of a weaker, vulnerable and marginalized group. The relatively low objective quality of life we find within the group that has tried cocaine (18.7 and 10.4%, respectively, below the groups that had not used drugs) is thought to support this hypothesis.

## Euphorics

Euphorics are drugs that are said to produce mental calmness, sedation and a euphoric detachment. An effective adaptation in the brain gives these drugs the capacity to create a strong physical dependence. The drugs are typically from the opiates and among regular users heroin is the drug of choice, because of the sudden high—“rush”—it gives when injected.

## Heroin / Morphine

Heroin use is widespread in Denmark and very strict prohibition has not had any effect in limiting its prevalence[20,21]. Indeed, prohibition may have increased the commercial attractiveness of the drug to the criminal underworld.

In Denmark, heroin and other opiates are typically used by young people to cope with existential discomfort. A parallel study in which we examined 50 heroin users showed that members of this group, in relation to the population in general, were more likely to have had a far worse background in terms of major traumatic events in childhood, such as neglect, abuse, and sexual abuse (unpublished data).

Of the 31 to 33 years old 2.8% had used heroin/morphine compared with 0.4% of the population sample (see Tables 1 and 2). Those 31 to 33 year olds that had used heroin/morphine, had a QOL score 13.5% below the cohort average, and for the population sample, those who had used heroin/morphine had a QOL score 9.8% below the population average, which is classified as an intermediate connection. Tables 3 and 4 show the connection is uniform through the eight QOL measures.

Presumably only a proportion of the 130 31 to 33 year olds that experimented with heroin/morphine developed dependence and heavy users are unlikely to answer a questionnaire. On the basis of the present research, it is difficult to make any conclusions with respect to the hypothesis that a few experiences with these drugs is detrimental to quality of life.

### Methadone

In Denmark methadone is distributed to registered drug addicts. Its prevalence on the black market is mainly due to heroin addicts selling these prescribed rations to buy heroin instead. 1.4% of the 31 to 33 year olds and 0.4% of the population sample had used methadone (see Tables 1 and 2). The 31 to 33 year olds were 16.6% below the mean QOL (see Table 3). Methadone users lie markedly low in objective quality of life, expressed in terms of social status and the wealth a person has attained—financial, physical and social.

### Polyuse

Polyuse is illustrated in this study by use of an example, namely, the combination of alcohol with diazepam/valium (a widely prevalent benzodiazepin). There is a powerful synergy between these two drugs; taken together they give a very strong intoxication. 4.7% of the 31 to 33 year olds had used this mixture (see Table 1) and the group had a QOL score 15.9% below the average (see Table 3).

This corresponded closely with methadone misuse, and we also observed a large overlap between these two groups. Multiple misuse is the cheapest form of chemical escape. It gives a powerful intoxication rather than a general feeling of euphoria and is thus an effective way of dealing with severe existential discomfort.

From the data it appeared that mixed drug use was just as damaging to QOL as heroin or morphine. However, it seems unlikely that the mixture of diazepam and alcohol, in itself, should be especially detrimental to quality of life. It is more probable that a low quality of life makes polyuse seem attractive. It is unlikely that legislation against the problem of multiple use could be enacted, as this would entail removing either alcohol or benzodiazepine from the Danish population.

## DISCUSSION

The use of conscious-altering drugs is widespread in Denmark. We found a small to intermediate correlation with quality of life (QOL). The connection was often a little stronger objectively than subjectively. Mixed use of socially acceptable drugs thought to be relatively harmless such as alcohol and diazepam (Valium) showed just as large a connection to quality of life and a similar prevalence as the so-called “hard” drugs, such as heroin, cocaine, and LSD. Most of the hallucinogenic drugs had a relatively weak connection to quality of life with cannabis having a very modest correlation. The stimulants showed the same modest connection. The depressants showed an intermediate and significant connection with quality of life. It should be considered whether the statistical connection is caused by adverse effects of the substance or through attempts to treat personal problems such as psychosis, low self-confidence, or existential discomfort.

It was found that approximately half of the Danish population has used illicit drugs. It seems that both mixing legal drugs to derive a similar effect as illegal drugs is widespread. By far the majority of these people have taken the drugs independently of illness, which presumably means that the use of drugs will not be discovered by the general practitioner in the normal course of a consultation.

Those that have used psychotropic drugs have, in general, a QOL score that is about 5% under the population average. There is a need for discussion among physicians and social welfare workers and agencies to determine what the practical consequences of this ought to be. However, in situations where a patient describes general feelings of discomfort it may be beneficial for the physician to approach the subject of drug use. It is important to admit that such a small difference in QOL could be more important than it seems, as there presumably is a strong statistical connection between QOL and functional ability in general. In a competitive society a loss of a few percent of a person’s functional ability can be important when it comes to job application, etc.

Of course one cannot claim that the use of psychotropic drugs is the cause of a low QOL. Quality of life is a very complex entity that involves all aspects of life such as attitudes, behavioural habits, relationships, understanding of life and self, and self-expression. It is developed throughout life from earliest childhood. It is therefore much more likely that a low quality of life encourages drug use. It seems highly relevant to think of the use of psychotropic drugs in terms of self-medication.

After diagnosis of a drug problem, as with other problems, it is tempting to take a direct approach and suggest reduced dosage regimens, detoxification with monitoring of blood and urine. The presented modest, albeit clear connection between use of drugs and quality of life suggests that working with quality of life and human relations in general may be a fruitful exercise. A better relationship to self, partner, family, work place or friends could, in most cases, lead to direct solutions of many of the problems underlying misuse.

From point of view of society and public health, serious consideration ought to be given to the prohibition of drugs. Evidently it does not have a considerable effect on the availability of drugs in the market place, and in many cases forces young people into a world of criminality and prostitution.

In our society resources might be spent more wisely on supporting the low QOL fractile of the population to a better QOL, instead of criminalizing the use of psychotropic drugs that in many cases looks more like justified self-medication for poor QOL than a criminal activity chosen by a healthy and sound person of free will.

## CONCLUSION

Experience of hallucinogenic and other psychotropic drugs is prevalent in the population and more the rule than the exception in the younger population. Only the use of euphoric drugs showed a significant connection of intermediate size with quality of life.

Since it is not clear whether the use of the drug itself leads to a poor quality of life or whether it is a poor quality of life that leads to self-medication with drugs, it is suggested that physicians in general practice and social welfare workers and agencies consider, together with detoxification and therapy, more general attempts to provide patients/clients with resources that may help them improve their qualities of life.

## ACKNOWLEDGEMENTS

This report presented results from the Danish Quality of Life Population Survey, the planning and execution of which would not have been possible without the aid of Dorte Loldrup Poulsen, Jørgen Hilden and [the late] Bengt Zachau-Christiansen. Funds were received from several Danish Foundations, including The 1991 Pharmacy, the Goodwill, the JL, E. Danielsens & Wife's, Emmerick Meyer's, the Frimodt-Heineken, the Family Hede Nielsen's, Petrus Andersen's, C.P. Frederiksen's and the Wedell-Wedellsborgs Foundations, and IMK Almene Fond. The research was approved by the Copenhagen Ethical Committee under numbers (KF) V 100.1762/90 and (KF) 01–502/93.

## BIOSKETCH

**Søren Ventegodt, MD,** is the director of the the Quality of Life Research Center in Copenhagen, Denmark. He is also responsible for a research clinic for holistic medicine in Copenhagen and used as a popular speaker throughout Scandinavia. He has published numerous scientific and popular articles and a number of books on holistic medicine, quality of life, and quality of working life. His most important scientific contributions are the comprehensive SEQOL questionnaire, the very short QoL5 questionnaire, the integrated QOL theory, the holistic process theory, the life mission theory, and the Danish Quality of Life Research Survey, 1991–94 in cooperation with the University Hospital of Copenhagen and the late pediatric professor Bengt Zachau-Christiansen. Website: www.livskvalitet.org/

**Joav Merrick, MD, DMSc,** is professor of child health and human development affiliated with the Zusman Child Development Center and Division of Community Health at the Ben Gurion University, Beer-Sheva, Israel and presently the medical director of the Division for Mental Retardation, Ministry of Social Affairs, Jerusalem and the director of the National Institute of Child Health and Human Development. He has authored and edited numerous publications in the fields of child and human development, rehabilitation, intellectual disability, disability, health, welfare, abuse, advocacy and prevention. Dr. Merrick received the Peter Sabroe Child Award for outstanding work on behalf of Danish Children in 1985 and the International LEGO-Prize ("The Children's Nobel Prize") for an extraordinary contribution toward improvement in child welfare and well-being in 1987. Website: www.nichd–israel.com
